# Engaging stakeholders to inform national implementation of critical time intervention in a program serving homeless-experienced Veterans

**DOI:** 10.3389/fpsyg.2022.1009467

**Published:** 2022-12-14

**Authors:** Sonya Gabrielian, Kristina M. Cordasco, Erin P. Finley, Lauren C. Hoffmann, Taylor Harris, Ronald A. Calderon, Jenny M. Barnard, David A. Ganz, Tanya T. Olmos-Ochoa

**Affiliations:** ^1^Health Services Research & Development (HSR&D) Center for the Study of Healthcare Innovation, Implementation and Policy (CSHIIP), Veterans Affairs (VA) Greater Los Angeles Healthcare System, Los Angeles, CA, United States; ^2^Desert Pacific Mental Illness Research, Education, and Clinical Center (MIRECC), VA Greater Los Angeles, Los Angeles, CA, United States; ^3^Department of Psychiatry and Biobehavioral Sciences, David Geffen School of Medicine, University of California, Los Angeles, Los Angeles, CA, United States; ^4^Department of Medicine, David Geffen School of Medicine, University of California, Los Angeles, Los Angeles, CA, United States; ^5^Division of Hospital Medicine, Department of Medicine and Department of Psychiatry, University of Texas Health San Antonio, San Antonio, TX, United States; ^6^Greater Los Angeles Geriatric Research, Education, and Clinical Center (GRECC), VA Greater Los Angeles, Los Angeles, CA, United States

**Keywords:** homelessness, Veterans, case management, implementation science, evidence-based practice

## Abstract

The Veterans Affairs (VA) Grant and Per Diem Case Management “Aftercare” program provides 6 months of case management for homeless-experienced Veterans (HEVs) transitioning to permanent housing, with the aim of decreasing returns to homelessness. Implementing Critical Time Intervention (CTI)—an evidence-based case management practice—would standardize care across the 128 community-based agencies that provide Aftercare services. To prepare for national CTI implementation in Aftercare, guided by Replicating Effective Programs (REP), we conducted a four-site pilot in which we adapted a CTI implementation package (training, technical assistance, and external facilitation); characterized stakeholder perspectives regarding the acceptability and appropriateness of this package; and identified contextual factors that affected CTI implementation. We engaged a stakeholder workgroup to tailor existing CTI training and technical assistance materials for Aftercare. To provide tailored support for providers and leaders to adopt and incorporate evidence-based practices (EBPs) into routine care, we also developed external facilitation materials and processes. Over 9 months, we implemented this package at four sites. We conducted semi-structured interviews at pre-implementation, mid-implementation, and 6 months post-implementation, with HEVs (*n* = 37), case managers (*n* = 16), supervisors (*n* = 10), and VA leaders (*n* = 4); these data were integrated with templated reflection notes from the project facilitator. We used rapid qualitative analysis and targeted coding to assess the acceptability and appropriateness of CTI and our implementation package and identify factors influencing CTI implementation. Stakeholders generally found CTI acceptable and appropriate; there was consensus that components of CTI were useful and compatible for this setting. To adapt our implementation package for scale-up, this pilot highlighted the value of robust and tangible CTI training and technical assistance—grounded in real-world cases—that highlights the congruence of CTI with relevant performance metrics. Variations in agency-level contextual factors may necessitate more intense and tailored supports to implement and sustain complex EBPs like CTI. Processes used in this pilot are relevant for implementing other EBPs in organizations that serve vulnerable populations. EBP scale-up and sustainment can be enhanced by engaging stakeholders to tailor EBPs for specific contexts; pilot testing and refining implementation packages for scale-up; and using qualitative methods to characterize contextual factors that affect EBP implementation.

## Introduction

Stable housing is a critical social determinant of health. Compared to their housed peers, homeless-experienced adults have worse behavioral health outcomes, higher prevalence of medical illness, and premature mortality (Dunn et al., 2006; Balshem et al., 2011; Carnemolla and Skinner, 2021; Paudyal et al., 2021; Onapa et al., 2022); these disparities are compounded by fragmented systems of care and discrimination experiences (Stafford and Wood, 2017; Ponka et al., 2020; Markowitz and Syverson, 2021; Schreiter et al., 2021). In the Department of Veterans Affairs (VA), ending homelessness among military Veterans in the United States of America is a national priority. Over the past decade, the VA made robust investments to scale-up Housing First (Tsemberis et al., 2004), an evidence-based practice (EBP) that pairs subsidies for permanent housing with field-based supportive services, which is often credited for a 50% decrease in Veteran homelessness (Henry et al., 2020). Veterans who remain homeless despite these advances are extraordinarily vulnerable; many live on the streets or are otherwise unsheltered and have mental illness and/or substance use disorders (Henry et al., 2021). To further VA's goal of ending Veteran homelessness, there is a pressing need to understand contextual factors that impact the scale up and spread of EBPs in settings that serve HEVs, and to develop effective practices that support such EBP implementation.

Implementation of Critical Time Intervention (CTI)—an evidence-based (Susser et al., 1997; Herman et al., 2000, 2011; Social Programs that Work, 2018; Ponka et al., 2020), structured, and time-limited case management practice—can substantively reduce returns to homelessness and decrease psychiatric hospitalizations among HEVs. Although CTI is an effective means of coordinating services for homeless adults, few HEVs receive CTI. To prepare for planned scale-up, spread, and sustainment of CTI in diverse community-based organizations that serve HEVs, we conducted a CTI implementation pilot in four agencies that partner with VA to serve HEVs. Over 9 months, this pilot was intended to adapt a CTI training, technical assistance, and external facilitation (Ritchie et al., 2014), an established process of providing tailored support for providers and leaders to adopt and incorporate EBPs into routine care. With a lens toward optimizing CTI scale up, this community case study describes processes used to adapt the CTI training and implementation supports; characterizes multi-level stakeholder perspectives regarding the acceptability and appropriateness of this package; and identifies contextual factors that affected CTI implementation.

## Context

The VA Grant and Per Diem (GPD) “Aftercare” program provides 6 months of case management for HEVs transitioning to permanent housing and not otherwise receiving case management, with the goal of decreasing returns to homelessness. This program launched in October 2019; services are provided by 128 community-based homeless service agencies across the nation that partner with VA to care for HEVs. Though Aftercare was designed to decrease HEVs' returns to homelessness, no specific case management paradigm is required in the program, resulting in significant practice variation across agencies.

Our policy partners at the GPD National Program Office identified CTI as an evidence-based, structured, and time-limited case management model that—if implemented nationally—would standardize and improve case management delivered in Aftercare. Our four-site implementation pilot aimed to inform plans for national implementation of CTI in Aftercare.

## Detail to understand key programmatic elements

**Figure 1:**
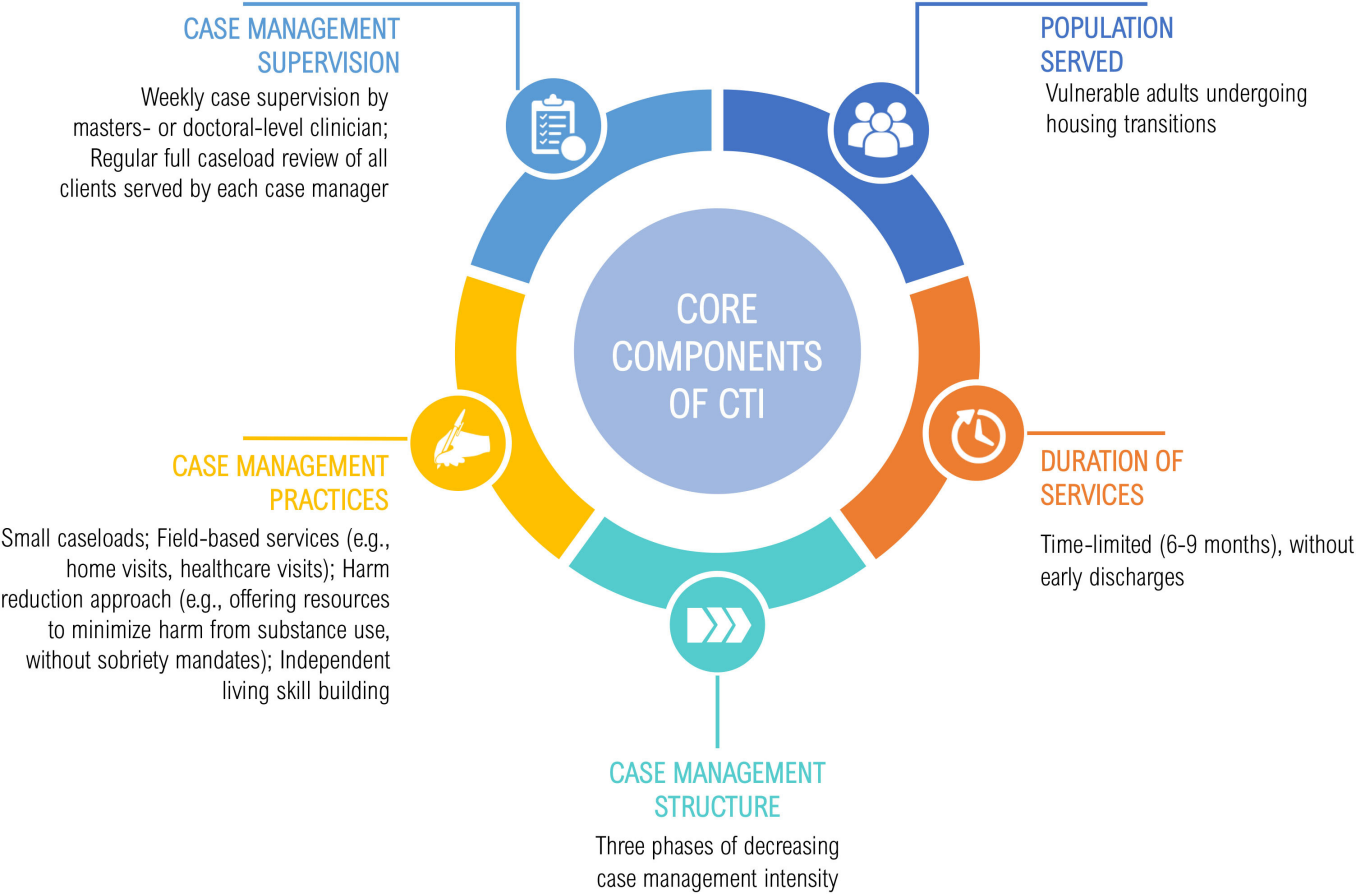
Core components of critical time intervention (CTI).

Figure 1 depicts the core components of CTI (Center for the Advancement of Critical Time Intervention, no date). Services are provided by a single case manager (“CTI specialist”) who delivers field-based services that help clients mobilize resources and support. Services are time-limited (6–9 months) and delivered in three phases of decreasing case management intensity. Using a harm reduction approach, CTI focuses on coordinating services and supports to enhance housing stability and meet clients' recovery goals, while building skills required for independent living (Social Programs that Work, 2018). Though CTI specialists have a range of backgrounds and training (ranging from consumer providers to clinicians with master's degrees), supervision practices are standardized, with a clinician who has master's-level training reviewing all clients served by each case manager on a weekly basis.

There is strong evidence, including five randomized controlled trials (RCTs) (Susser et al., 1997; Herman et al., 2000, 2011; Social Programs that Work, 2018) and a systematic review (Ponka et al., 2020), that CTI improves housing stability and decreases psychiatric hospitalizations among homeless-experienced adults. Moreover, CTI was successfully implemented in 8 VA facilities for HEVs with serious mental illness, suggesting it is feasible and appropriate for scale up and spread within VA (Kasprow and Rosenheck, 2007), the nation's largest provider of services for homeless adults, many of whom have serious mental illness or other behavioral health disorders. However, little is known about strategies that support the implementation of complex case management practices in diverse community-based organizational settings that serve homeless adults. This implementation pilot aimed to fill these gaps, preparatory to a subsequent national implementation initiative. All pilot activities received a determination of non-research by the VA Central Institutional Review Board.

### Adapting a CTI implementation package

Initial development of the CTI implementation package for Aftercare was guided by the Replicating Effective Programs (REP) framework (Kilbourne et al., 2014; Hamilton et al., 2017), which uses stakeholder input to enable packaging, training, and technical assistance of EBPs. REP was intended to enhance case managers' CTI skills and clinical competency, thereby enhancing CTI implementation.

Though REP has four phases (pre-conditions; pre-implementation; implementation; and maintenance and evolution) in total, only the first two apply to this implementation pilot (detailed in Table 1). Phases 3 and 4 will be encompassed in the planned national scale-up.

**Table 1:** Phases 1 and 2 of REP specified for a CTI implementation pilot in Aftercare.

**Phase**	**Process**	**Products**
I: Pre-conditions	• Select CTI as an EBP in partnership with national policy partners • Identify implementation barriers to CTI in Aftercare • Build a stakeholder workgroup that identifies CTI core components and adaptation options	• List of CTI's core components • Menu of options to adapt CTI for Aftercare
II: Pre-implementation	• Assemble CTI training, technical assistance, and implementation support package with stakeholder workgroup • Orient Aftercare program staff to CTI and plan for logistics • Pilot test and refine the CTI implementation package at four Aftercare sites	• Refined CTI implementation package for national scale-up and spread

In Phase 1 (pre-conditions), we selected CTI in partnership with our policy partners and assembled a seven-member virtual stakeholder workgroup, comprised of CTI practitioners and trainers, local and national Aftercare clinicians and leaders, and HEVs. This group held four videoconference sessions (2 h each) to tailor, for the Aftercare context, a CTI training and technical assistance package recently implemented in homeless programs in Connecticut for homeless-experienced civilians (Critical Time Intervention/Rapid Re-housing Pilot, 2017).

First, the workgroup reached consensus on CTI's theory of change, i.e., the practice's core components (Figure 1). Next, we made practice adaptations to reflect the Aftercare context; for example, as case manager engagement with HEVs before Aftercare enrollment (“pre-CTI”) is programmatically difficult, typical pre-CTI processes (e.g., gathering psychosocial data, establishing key recovery goals) were shifted to the first phase of CTI. As HEVs have higher rates of trauma and less social support than their homeless-experienced, non-Veteran peers (Tsai and Rosenheck, 2015), principles of trauma-informed care and social skill building, respectively, were included in CTI training and practice. Clinical vignettes presented in CTI training and technical assistance materials were adapted to reflect the diverse social circumstances, functioning, and diagnoses of HEVs in Aftercare. Additional adaptations were made in response to public health precautions imposed by the Coronavirus disease 2019 (COVID-19) pandemic, including the inclusion of virtual case management practices and strategies to address the “digital divide” that can impede health information access by vulnerable populations (Eruchalu et al., 2021). Additionally, all training and technical assistance materials were adapted for videoconference and/or in-person delivery, including an online toolkit and training slide decks.

In Phase 2 of REP, the final CTI implementation package (Table 2) was pilot tested at four Aftercare sites. This package consisted of: an intensive CTI training (six synchronous videoconference sessions led by expert CTI trainers); monthly communities of practice (CoP), i.e., one-hour discussions to deepen knowledge and expertise in CTI, attended by case managers and supervisors across all implementing Aftercare sites *via* synchronous videoconference; and on-demand telephone or videoconference case consultation with a CTI-trained clinician with expertise in HEVs. In addition, we developed external facilitation materials and processes; a facilitator trained in CTI and implementation facilitation provided tailored support *via* biweekly 30-min videoconferences with each site. These sessions aimed to build sites' organizational capacity to implement CTI and empower case managers to enact systems-based change that promotes CTI implementation (Lessard et al., 2016). The facilitator completed a templated reflection form after each call that included a summary of the call, successes of and challenges to facilitation, implementation strategies employed, and next steps.

**Table 2:** CTI implementation package piloted at four Aftercare sites.

**Component**	**Description**	**Delivery time**
Intensive CTI training	Six synchronous videoconference sessions (2 h/week for 6 weeks)	Once, at the start of CTI implementation
Community of Practice (CoP) Sessions	Synchronous videoconferences to deepen knowledge and expertise in CTI, anchored in a brief presentation by the CoP leader or a guest speaker, followed by moderated interaction among Aftercare case managers and supervisors (1 h each)	Monthly, for 6 months, starting the month after the 6-session intensive CTI training is complete
On-demand case consultation with a CTI expert	Telephone call or synchronous videoconference to discuss an Aftercare case, with consultation grounded in fidelity to CTI (30 min each)	As needed by any Aftercare staff, throughout the 9-month implementation pilot
External facilitation	Implementation- and support-oriented activities, delivered *via* synchronous videoconference, tailored for each site (30 min each)	Every 2 weeks, for 6 months, starting the month after the 6-session intensive CTI training is complete

### Key stakeholder interviews to assess CTI implementation

A team of trained qualitative analysts conducted a total of 67 semi-structured telephone interviews (45 min each) across three time points: baseline (pre-implementation) and three- and six-months post-CTI implementation. Interviews were conducted with HEVs (*n* = 37) at baseline only. We interviewed Aftercare case managers (*n* = 16), supervisors (*n* = 10), and VA leadership (*n* = 4) across the four pilot sites at all three time points. We obtained verbal consent for all interviews. We provided confidentiality and privacy assurances as part of the consent process; interviews were analyzed in aggregate and all information linking individuals to interview data was destroyed prior to analyses.

Interviews with HEVs assessed their perceived needs and care experiences in Aftercare. Case manager and supervisor interviews were grounded in the Consolidated Framework for Implementation Research (CFIR) (Damschroder et al., 2009), which consolidates constructs across a breadth of implementation science frameworks (Damschroder et al., 2009; Damschroder and Hagedorn, 2011) and is well suited to characterize factors influencing implementation outcomes. Baseline interviews assessed staff background and training, case management practices, and factors pertaining to the inner setting (organizational context) and outer setting (socioeconomic and political context) that might affect implementation success. Three- and six-month interviews characterized experiences with CTI training, perspectives regarding CTI's acceptability and appropriateness, and recommendations to enhance CTI implementation support.

Baseline interviews with VA leaders assessed their prior knowledge of CTI and assessment of CTI's general fit with Aftercare. At follow-up, VA leaders were asked to evaluate participating sites' CTI implementation and to make recommendations for improving the CTI implementation package. Of note, we included additional contextual data from the facilitator's reflections (*n* = 44), focused on her interpretations of each site's implementation successes and challenges.

All interviews were audio recorded and professionally transcribed. Using rapid qualitative analysis methods (Abraham et al., 2021), we created structured summaries of each interview organized by interview question and/or CFIR domains; and summaries of each facilitation session highlighting successes and challenges. We also created summaries by key stakeholder and implementing site (e.g., HEV perspectives from Site 1). We assessed satisfaction with CTI and our implementation package, as well as contextual factors (at the organizational or program level) influencing CTI implementation. We then conducted targeted in-depth coding using ATLAS.ti software, assessing the acceptability and appropriateness of CTI and our implementation package, and identifying factors influencing CTI implementation that were relevant for informing the planned national scale-up.

### Acceptability and appropriateness of the CTI implementation package in Aftercare

CTI's core components were aligned with multi-level stakeholders' needs and goals. HEVs' stated goals for the Aftercare program were congruent with CTI principles, e.g., financial stability (*via* rental assistance, income and other benefits, budgeting), and engaging with mental and physical health care, employment, and legal assistance. VA leadership viewed CTI implementation as an opportunity to standardize and improve case management in Aftercare. Though case managers and supervisors had limited prior experience implementing EBPs, they desired case management training that was grounded in real-world cases. As one supervisor asserted, “*I want something tangible and realistic…I don't want another training on how to put a [Veteran's case] file together. I don't want anyone telling me the basics of case management.”* Supervisors also desired clarity regarding Aftercare performance metrics and standardization of case management processes.

Overall, Aftercare staff were highly satisfied with the CTI training and found the content straightforward and helpful, albeit similar to other case management trainings. Most Aftercare case managers and supervisors had no knowledge of CTI prior to the intensive training. Post-training, case managers and supervisors at all sites described having some components of CTI in place at their organizations prior to the pilot; they believed that successful CTI implementation would require simple changes to existing processes: As described by a supervisor, “*For the most part, we were already doing the majority of [CTI] already, so it was a pretty seamless transition.”* Nonetheless, case managers and supervisors requested more opportunities to share practices and to engage in asynchronous learning; they also desired ongoing CTI training refreshers from a knowledgeable CTI trainer to strengthen newly acquired practice knowledge and to clarify content.

Case managers and supervisors described CTI as providing needed structure to case management practice and building case managers' skills. As one case manager stated, “*I really like that there's the three stages, two months each. I think it's a good way to organize the work that can be done with the Vets*.”

Nonetheless, at 6 months post-implementation, all sites remained uncertain about how to implement specific CTI components, e.g., adapting clinical supervision practices and case consultation to support CTI adoption. Three of the four sites described limited buy-in for CTI's goal-focused and time-limited case management; these sites remained unconvinced throughout the pilot that a six-month case management practice was sufficient time to address the significant psychiatric, medical, and social needs of HEVs on their caseloads. Across the four sites, by six months post-implementation, case managers and supervisors felt that CTI was sufficiently implemented but that they needed more time for CTI to “*become second nature”* and to characterize factors relevant to its sustainment after the pilot's implementation supports ceased.

External facilitation targeted many of the sites' stated concerns about CTI's acceptability and appropriateness. The facilitator was instrumental in highlighting the differences between the sites' existing case management practices and CTI; the facilitator supported sites in making adaptations to CTI to fit their local contexts, while maintaining practice fidelity. Though Aftercare services are for 6 months, many agencies providing Aftercare were accustomed to long-term case management. CTI's core differences derived from its: time-limited nature; focus on recovery goals connected to HEVs' history of housing instability; and emphasis on care coordination. Transitioning to CTI case management required a shift in case managers' and supervisors' conceptualization of their roles and functions (i.e., redefining how successful case management looks under CTI) and the routinization of key CTI components into everyday case management practice (e.g., setting-focused recovery goals achievable in 2–6 months). As such, this implementation pilot highlighted areas that were insufficiently addressed by CTI training and technical assistance and required more robust support from facilitation.

### Contextual factors impacting implementation across all pilot sites

The COVID-19 pandemic began shortly after the launch of the Aftercare program, with significant logistical impacts on CTI implementation. The pandemic led to: changes in Aftercare work structure (e.g., reduced in-home visits, increased telework); increased challenges coordinating services with VA and community-based agencies due to closures and staffing shortages; heightened barriers to stated recovery goals (e.g., finding employment, establishing mental health and medical services); and Aftercare staff burnout and turnover. As stated by a case manager, “*You are only as good as your resources. Once those are gone, you are doing things as creatively as you can.”* These feelings were echoed by another case manager who said, “*There are some referrals where there is very little accommodation. They are your last shot, and they hang up. There are others where…they may say they'll talk to the Veteran and refer them [back] to you for additional services... There are varying degrees of how successful those warm handoffs are.”*

In addition, all sites reported concerns complying with national Aftercare requirements related to HEVs' eligibility for the program (e.g., enrollment criteria described as too stringent) and HEV recruitment (e.g., perceived competition with other Aftercare sites for HEVs). As stated by a supervisor, “*We've been all over the [VA] campus letting them know that we're there and we're ready to take referrals. According to our case manager he's been met with some resistance because [the VA] provides some case management as well.”*

Across sites, case managers and supervisors expressed significant uncertainty about how “success” would be measured in Aftercare. There was a lack of clarity surrounding Aftercare's core functions; while there was an accepted programmatic aim to decrease returns to homelessness, key case management tasks that would enable this aim were often vaguely conceptualized. Few, if any, quality or performance metrics were conveyed by national leadership to staff at these pilot sites, leading to confusion about best case management practices to employ.

Staffing instability due to case manager and supervisor turnover at all sites resulted in varying degrees of CTI adoption among remaining staff. Two sites experienced supervisor turnover; one site was unable to identify a replacement during the pilot period and the other appointed an interim supervisor with limited slack. At times, supervisor turnover led to periods without clinical supervision, slowing case managers' supports and motivation to implement CTI. The remaining two sites experienced case manager turnover and/or illness requiring extended leave. Remaining staff often took over HEVs' cases managed by staff who left; at some sites, remaining staff also assumed responsibilities for training and onboarding new staff. For some case managers, increased administrative and clinical demands resulting from staff turnover limited their opportunity and motivation to adopt CTI.

### Site-specific contextual factors influencing implementation

Contextual factors influencing CTI implementation also varied by site. Table 3 summarizes site-specific contextual factors that challenged implementation success. There were variations in case managers' backgrounds and training; some case managers were master's level clinicians (e.g., in family therapy or social work) and others were transitioning from other disciplines or were recent college graduates with limited case management experience. This breadth of backgrounds and training led to, at some sites, challenging case manager-supervisor dynamics. Less experienced case managers often were highly dependent on case consultation and clinical supervision. Beyond interpersonal dynamics, CTI implementation was also influenced by leadership buy-in, competing staff responsibilities, caseload sizes, geography and resource limitations, as well as incongruence between case manager beliefs and components of CTI practice.

**Table 3:** Summary of contextual factors that challenged CTI implementation at each pilot site.

**Pilot site**	**Key contextual factors**
Site 1	• Limited leadership engagement in CTI implementation • Lack of structured training and onboarding of the site's single case manager • Challenging team dynamics • Significant challenges recruiting HEVs into Aftercare
Site 2	• Organizational pressures to have large Veteran caseloads • Resistance to the six-month duration of the Aftercare program (deemed too brief for the complexity of HEVs enrolled)
Site 3	• Staffing shortages led to competing staff responsibilities within the agency but outside the Aftercare program • Engaged supervisor and well-functioning team • Case managers desired to provide psychotherapy and other clinical services, rather than focus on the CTI's core care coordination practices
Site 4	• Transient caseloads (HEVs often move out of the site's catchment area) • Long commutes are required for field visits that span two counties • More limited community-based resources and referrals than the pilot's urban sites • Supervisor turnover resulted in higher case consultation and facilitation needs

Site 1 struggled with significant challenges recruiting HEVs into its program, poor leadership engagement in CTI implementation, and challenging team dynamics. The new and only case manager received minimal training at onboarding and was overwhelmed establishing relationships with VA and non-VA stakeholders to recruit HEVs to Aftercare. From the perspective of VA leadership, Site 1 struggled due to chronically small caseloads; in fact, this site had a caseload of zero HEVs for an extended period during our pilot, bringing CTI implementation to a standstill while the case manager focused on recruitment. Stakeholders at multiple levels described insufficient support from site leadership for CTI adoption. Our external facilitator recounted that Site 1 presented the most difficult team dynamics between the case manager and supervisors. The case manager verbalized needing supervisor support but was not always receptive to supervisor input.

Stakeholders at Site 2 struggled with high caseloads and some misalignment between staff beliefs and CTI principles. Case managers at this site described the most initial resistance to CTI, with the senior case manager remarking that 6 months was insufficient time to build rapport with HEVs and link them to necessary resources. As stated by a site supervisor, “*Six months doesn't seem to be long enough, if someone has a habit, they have had all their life, it's really hard to change it.”* This site struggled to engage leadership in the implementation pilot and described organizational pressures to enroll more HEVs onto their caseloads, leaving case managers overwhelmed. Interview data suggested that addressing resistance to CTI may have benefitted from an in-person meeting with our implementation team (meetings were held virtually due to physical distancing precautions of the COVID-19 pandemic) and more intense external facilitation support.

Site 3 exhibited strong team dynamics but struggled with staffing shortages and challenges differentiating CTI practice from their baseline case management. Moreover, its experienced case managers expressed resistance to CTI's salient care coordination practices, asserting that linking HEVs to longitudinal resources without addressing their needs up front was inadequate case management (e.g., rather than linking a HEV to mental health services, these case managers wanted to provide psychotherapy themselves). Case managers and supervisors struggled to differentiate CTI's core components from baseline practice, challenging practice fidelity. Additionally, due to medical leave and staffing shortages, case managers were given additional duties that were outside the scope of Aftercare, which delayed CTI implementation.

Site 4 had the most unique challenges related primarily to its geographic location, compounded by supervisor turnover. As the site's case manager described, “*Unlike urban or dense [population] programs, we are covering two counties with one staff member… to get to a client, it can take 1 to 2 h on the highway.”* The case manager and supervisor described HEVs on their caseload as transient; the case manager had to intentionally assess how familiar HEVs were with the area before linking them to resources. Long drives (>1 h) to and from HEVs' homes challenged the case manager's ability to connect with HEVs at the appropriate intensity for CTI. The case manager expected these challenges to worsen as the number of HEVs in the program increased. It was also difficult for the case manager to “keep up” with changing community resources and network with other organizations over such a large geographic area. Despite these challenges, site stakeholders were motivated to use CTI, but required more case consultation and tailored implementation support from the facilitator when the supervisor left the organization.

## Discussion

We conducted a pilot project to implement CTI in four community-based agencies that provide time-limited case management services for HEVs as part of VA's Aftercare program. This pilot was a valuable opportunity to assess early CTI implementation outcomes in different Aftercare settings and contexts; overall, Aftercare stakeholders found CTI acceptable and appropriate. There was consensus that components of CTI were compatible and useful for this setting, despite some concerns that remained salient throughout implementation. As we moved toward adapting our CTI implementation package for national scale-up, our findings highlighted the value of robust and tangible CTI training and technical assistance—grounded in real-world Veteran cases—that highlights the congruence of CTI with relevant VA performance metrics. Moreover, our data suggest that variations in agency-level contextual factors may necessitate more intense and tailored supports (e.g., external facilitation, case consultation, learning collaboratives) to implement and sustain complex EBPs like CTI. Table 4 summarizes key adaptations to our implementation package (i.e., CTI training, technical assistance, and external facilitation) for the national initiative that derives from this pilot. We anticipate that these adaptations will enhance key outcomes in the planned national implementation initiative, including CTI fidelity and sustainment. As CTI's effectiveness is influenced by fidelity to its core components, we also hypothesize that these adaptations will influence important quality metrics (e.g., housing stability, hospitalization rates, and HEV and case manager experiences).

**Table 4:** Adaptations to CTI implementation package in preparation for national scale-up.

**Package component**	**Adaptations for national scale-up**
CTI training and technical assistance	•Clarify key differences between CTI and traditional case management for HEVs
	• Enhance CTI training materials with more Veteran and VA-focused examples, including Veterans with psychiatric and medical complexities
	• Develop an online CTI toolkit as a central repository for CTI resources (e.g., CTI manual, progress note templates)
	• Develop an e-mail listserv to facilitate shared learning
	• Increase the frequency of CTI community of practice sessions, led by an experienced moderator (a CTI trained licensed clinical social worker leading sessions twice a month, up from monthly during the pilot)
	• Develop CTI “refresher sessions” to enhance practice sustainment when implementation supports cease
	• Foster shared learning among sites about successful recruitment practices
	• Develop a system to onboard new case managers and supervisors to CTI (given likelihood of staff turnover during implementation)
External facilitation	• Increase frequency of external facilitation calls (from biweekly to weekly)
	• Engage case managers and supervisors in early conversations about recruitment practices during external facilitation sessions
	• Use early facilitation calls to develop a structured site profile (e.g., site geography, staffing challenges, available resources, relationships with VA providers, knowledge of VA resources) to aid with implementation
	• Set realistic organizational expectations about caseload size, derived from CTI fidelity measures
	• Engage leadership early and often as part of external facilitation
	• Clarify agency and program-level performance metrics and support sites in using CTI to meet these metrics

These methods and findings have relevance for implementing other multi-faceted EBPs in diverse community-based organizations that serve homeless-experienced adults. While a breadth of EBPs (Munthe-Kaas et al., 2018; Pottie et al., 2020; Lowman and Sheetz, 2021; Semborski et al., 2021) [e.g., Housing First, harm reduction paradigms, and Assertive Community Treatment (multidisciplinary, team-based case management approach with assertive community outreach)] effectively address homelessness among adults with behavioral health disorders, it is immensely challenging to implement and sustain such practices with fidelity (Casey et al., 2013; Smelson et al., 2022; Tidmarsh et al., 2022). In this pilot, we used REP (Kilbourne et al., 2014; Hamilton et al., 2017) to engage multi-level stakeholders in a structured approaches to tailor an EBP for a specific context. We were able to refine training and implementation supports for this EBP using qualitative data that highlighted contextual factors that supported or impeded EBP implementation. Specifically, consistent with Phases 1 and 2 of REP, we performed key steps (Kilbourne et al., 2014; Hamilton et al., 2017) that can be used across practices and settings to prepare EBPs for effective scale-up and spread: (1) assembled a stakeholder workgroup to identify the core components of an EBP and its adaptation options; (2) tailored the EBP, focused on its training and implementation supports, for the setting and context; (3) pilot tested the tailored EBP and its implementation supports; (4) used qualitative methods to gather stakeholder perspectives on the EBP and its implementation in the pilot; and (5) engaged in data-informed adaptations and refinements to the EBP. This approach is critical to ensure that complex EBPs—and their implementation supports (e.g., training, technical assistance, facilitation)—are optimally tailored and formalized prior to planned scale-up. These efforts can enable greater fidelity and sustainment, as well as effectiveness, in larger implementation initiatives. Though the implementation of EBPs requires a careful balance between tailoring interventions to contexts and maintaining fidelity to an EBP's core components (Von Thiele Schwarz et al., 2021; Wiltsey Stirman, 2022), we highlight the value of pilot work that tailors and enhances EBP training, technical assistance, and implementation supports to reflect relevant contextual factors.

Our data highlight the diversity of organizational characteristics and other contextual factors likely to support or impede EBP implementation for vulnerable populations. Consistent with a systematic review (Valenstein-Mah et al., 2020) that concluded that EBP training in isolation improves short-term provider satisfaction and EBP knowledge, but does not impact provider knowledge, we found that some Aftercare providers require more intense and costly supports to achieve CTI adoption. Yet, many community-based mental health and social service agencies that serve homeless-experienced persons rely heavily on EBP training alone, with clinical supervision, to enhance provider training and knowledge, as well as client outcomes. Particularly given profound deficits in community-based homeless service providers' workforce wellbeing, with high rates of burnout and turnover (Rollins et al., 2010; Salyers et al., 2013; Sullivan et al., 2015; Wirth et al., 2019; Peters et al., 2022), ensuring ample supports (e.g., training, technical assistance, facilitation) for EBP adoption is critical.

To date, little is known about organizational structures and characteristics, within and beyond homeless service agencies, that interact with provider capabilities, opportunity, and motivation, to influence EBP implementation (Michie et al., 2011; Mather et al., 2022). To fill this gap, our planned national implementation initiative will use a cluster randomized design to compare the implementation and effectiveness of two approaches to support CTI implementation across sites: CTI training and technical assistance alone (base implementation strategy) vs. CTI training and technical assistance enhanced by external facilitation (enhanced implementation strategy).

## Acknowledgment of any conceptual or methodological constraints

This implementation pilot is limited by its focus on four community-based organizations that serve HEVs. These organizations, and the HEVs they serve, may differ from other Aftercare sites and HEVs, respectively, in other geographic regions. Of note, our findings are most applicable for organizations that partner with VA to serve HEVs; they may not extrapolate to other organizations and homeless populations. However, we suspect that methods used for this implementation pilot may benefit other EBP implementation initiatives for populations of homeless-experienced adults who do not use VA.

As a nine-month pilot at four sites, conducted during the COVID-19 pandemic, we were limited by our reliance on semi-structured interviews conducted by phone; we were unable to augment these data with site visits or in-person data collection with vulnerable HEVs who may not have access to phones. Moreover, aligned with our primary goal to adapt our implementation supports (training, technical assistance, and facilitation) for scale up, we intentionally focused our qualitative data collection efforts on providers; as such, we only interviewed HEVs at baseline. Follow-up interviews with HEVs, which we plan to conduct in the national implementation initiative, would provide critical information about how HEVs perceive the core components of CTI, as well adaptations to the practice that derived from our stakeholder workgroup. In addition, given the goals and scope of this project, the rich narratives provided by our semi-structured interview data allowed for a nuanced understanding of contextual factors that affected CTI implementation; however, additional structured data collection, e.g., structured and validated assessments of practice acceptability, would enhance our findings.

Of note, due to our project's sample size and timeline, we were unable to gather data about CTI's effectiveness as part of this pilot initiative; as CTI is a well-established EBP (Susser et al., 1997; Herman et al., 2000, 2011; Social Programs that Work, 2018; Ponka et al., 2020; Weightman et al., 2022), we relied heavily on existing data about practice effectiveness. However, the planned national implementation initiative will collect data about CTI's implementation and effectiveness by integrating qualitative and quantitative data.

## Conclusions

CTI was successfully implemented in four agencies that provide Aftercare services for HEVs. This pilot used REP to inform adaptation, piloting, and refinement of a CTI implementation package that will be used in a national implementation initiative. Our data is well-aligned with literature suggesting that implementing EBPs in diverse settings requires balancing practice fidelity with adaptations that accommodate contextual differences across settings (Chambers et al., 2013; Reed et al., 2018; Wiltsey Stirman et al., 2019). At some agencies, longitudinal implementation supports may be important to address key contextual characteristics that interplay with behavioral change factors (Michie et al., 2011) (i.e., capability, opportunity, or motivation) to influence CTI implementation. We plan to test more intense supports—and evaluate whether specific contextual factors are more likely to require such supports to implement and sustain CTI—in the planned national implementation initiative.

## Data availability statement

All relevant data is contained within the article. The original contributions presented in the study are included in the article, further inquiries can be directed to the corresponding author.

## Ethics statement

The studies involving human participants were reviewed and approved by VA Central Institutional Review Board as a quality improvement activity. Written informed consent for participation was not required for this study in accordance with the national legislation and the institutional requirements.

## Author contributions

SG, KC, EF, DG, and TO-O contributed to the conception and design of this project. LH wrote sections of the manuscript. TH, RC, and JB contributed to data collection and analyses. All authors contributed to manuscript revision and read and approved the submitted version.

## References

[B1] Abraham T. H. Finley E. P. Drummond K. L. Haro E. K. Hamilton A. B. Townsend J. C.. (2021). A method for developing trustworthiness and preserving richness of qualitative data during team-based analysis of large data sets. Am. J. Eval. 42, 139–156. 10.1177/1098214019893784

[B2] Balshem H. Christensen V. Tuepker A. Kansagara D. (2011). A Critical Review of the Literature Regarding Homelessness Among Veterans. VA Evidence-based Synthesis Program Reports. Washington, DC: Department of Veterans Affairs (US). 21678634

[B3] Carnemolla P. Skinner V. (2021). Outcomes Associated with Providing Secure, Stable, and Permanent Housing for People Who Have Been Homeless: An International Scoping Review. J. Plan. Lit. 36, 508–525. 10.1177/08854122211012911

[B4] Casey R. Clark C. Smits P. Peters R. (2013). Application of implementation science for homeless interventions. Am. J. Public Health 103(Suppl. 2), S183–S184. 10.2105/AJPH.2013.301729 24148045 PMC3969144

[B5] Center for the Advancement of Critical Time Intervention (no date). CTI Model. Available online at: www.Criticaltime.org/cti-model/ (accessed April 19 2021).

[B6] Chambers D. A. Glasgow R. E. Stange K. C. (2013). The dynamic sustainability framework: addressing the paradox of sustainment amid ongoing change. Implement. Sci. 8, 117. 10.1186/1748-5908-8-117 24088228 PMC3852739

[B7] Critical Time Intervention/Rapid Re-housing Pilot (2017). Available online at: https://cceh.org/cti-rrh/ (accessed April 20, 2021).

[B8] Damschroder L. J. Aron D. C. Keith R. E. Kirsh S. R. Alexander J. A. Lowery J. C. (2009). Fostering implementation of health services research findings into practice: a consolidated framework for advancing implementation science. Implement. Sci. 4, 50. 10.1186/1748-5908-4-50 19664226 PMC2736161

[B9] Damschroder L. J. Hagedorn H. J. (2011). A guiding framework and approach for implementation research in substance use disorders treatment. Psychol. Addict. Behav. 25, 194–205. 10.1037/a0022284 21443291

[B10] DunnJ. R.HayesM. V.HulchanskiJ. D.HwangS. W.PotvinL. (2006). Housing as a socio-economic determinant of health: findings of a national needs, gaps and opportunities assessment. Ca.n J. Public Health 97 (Suppl 3), S11-5, S12–S17. 10.1007/BF0340539217357542

[B11] Eruchalu C. N. Pichardo M. S. Bharadwaj M. Rodriguez C. B. Rodriguez J. A. Bergmark R. W.. (2021). The expanding digital divide: digital health access inequities during the COVID-19 Pandemic in New York City. J. Urban Health 98, 183–186. 10.1007/s11524-020-00508-9 33471281 PMC7816740

[B12] Hamilton A. B. Farmer M. M. Moin T. Finley E. P. Lang A. J. Oishi S. M.. (2017). Enhancing Mental and Physical Health of Women through Engagement and Retention (EMPOWER): a protocol for a program of research. Implement. Sci. 12, 127. 10.1186/s13012-017-0658-9 29116022 PMC5678767

[B13] Henry M. De Sousa T. Roddey C. Gaven S. Bednar T. (2021). The 2020 Annual Homeless Assessment Report (AHAR) to Congress. Available online at: https://www.huduser.gov/portal/sites/default/files/pdf/2020-AHAR-Part-1.pdf (accessed September 1, 2022).

[B14] Henry M. Watt R. Rosenthal L. Shivii A. (2020). The 2020 Point-in-Time Estimates of Homelessness: Part I of the 2020 Annual Homeless Assessment Report to Congress. Available online at: https://www.huduser.gov/portal/datasets/ahar/2020-ahar-part-1-pit-estimates-of-homelessness-in-the-us.html (accessed September 1, 2022).

[B15] Herman D. Conover S. Gorroochurn P. Hinterland K. Hoepner L. Susser E. (2011). Randomized trial of critical time intervention to prevent homelessness after hospital discharge. Psychiatr. Serv. 62, 713–719. 10.1176/ps.62.7.pss6207_0713 21724782 PMC3132151

[B16] Herman D. Opler L. Felix A. Valencia E. Wyatt R. J. Susser E. (2000). A critical time intervention with mentally ill homeless men: impact on psychiatric symptoms. J. Nerv. Ment. Dis. 188, 135–140. 10.1097/00005053-200003000-00002 10749277

[B17] Kasprow W. J. Rosenheck R. A. (2007). Outcomes of critical time intervention case management of homeless veterans after psychiatric hospitalization. Psychiatr. Serv. 58, 929–935. 10.1176/ps.2007.58.7.929 17602008

[B18] Kilbourne A. M. Almirall D. Goodrich D. E. Lai Z. Abraham K. M. Nord K. M.. (2014). Enhancing outreach for persons with serious mental illness: 12-month results from a cluster randomized trial of an adaptive implementation strategy. Implement. Sci. 9, 163. 10.1186/s13012-014-0163-3 25544027 PMC4296543

[B19] Lessard S. Bareil C. Lalonde L. Duhamel F. Hudon E. Goudreau J.. (2016). External facilitators and interprofessional facilitation teams: a qualitative study of their roles in supporting practice change. Implement. Sci. 11, 97. 10.1186/s13012-016-0458-7 27424171 PMC4947272

[B20] Lowman C. A. Sheetz R. L. (2021). VA Clinical Services: The Key to Achieving Stability and Sustainment for Homeless Veterans. Clinical Management of the Homeless Patient. Springer. 10.1007/978-3-030-70135-2_20

[B21] Markowitz F. E. Syverson J. (2021). Race, gender, and homelessness stigma: effects of perceived blameworthiness and dangerousness. Deviant Behav. 42, 919–931. 10.1080/01639625.2019.1706140

[B22] Mather M. Pettigrew L. M. Navaratnam S. (2022). Barriers and facilitators to clinical behaviour change by primary care practitioners: a theory-informed systematic review of reviews using the Theoretical Domains Framework and Behaviour Change Wheel. Syst. Rev. 11, 180. 10.1186/s13643-022-02030-2 36042457 PMC9429279

[B23] Michie S. Van Stralen M. M. West R. (2011). The behaviour change wheel: a new method for characterising and designing behaviour change interventions. Implement. Sci. 6, 42. 10.1186/1748-5908-6-42 21513547 PMC3096582

[B24] Munthe-Kaas H. M. Berg R. C. Blaasvær N. (2018). Effectiveness of interventions to reduce homelessness: a systematic review and meta-analysis. Campbell Syst. Rev. 14, 1–281. 10.4073/csr.2018.3 37131370 PMC8427990

[B25] Onapa H. Sharpley C. F. Bitsika V. Mcmillan M. E. Maclure K. Smith L.. (2022). The physical and mental health effects of housing homeless people: a systematic review. Health Soc. Care Community 30, 448–468. 10.1111/hsc.13486 34423491

[B26] Paudyal V. Ghani A. Shafi T. Punj E. Saunders K. Vohra N.. (2021). Clinical characteristics, attendance outcomes and deaths of homeless persons in the emergency department: implications for primary health care and community prevention programmes. Public Health 196, 117–123. 10.1016/j.puhe.2021.05.007 34182257

[B27] Peters L. Hobson C. W. Samuel V. (2022). A systematic review and meta-synthesis of qualitative studies that investigate the emotional experiences of staff working in homeless settings. Health Soc. Care Community 30, 58–72. 10.1111/hsc.13502 34255385

[B28] Ponka D. Agbata E. Kendall C. Stergiopoulos V. Mendonca O. Magwood O.. (2020). The effectiveness of case management interventions for the homeless, vulnerably housed and persons with lived experience: a systematic review. PLoS ONE 15, e0230896. 10.1371/journal.pone.0230896 32271769 PMC7313544

[B29] Pottie K. Kendall C. E. Aubry T. Magwood O. Andermann A. Salvalaggio G.. (2020). Clinical guideline for homeless and vulnerably housed people, and people with lived homelessness experience. CMAJ 192, E240–E254. 10.1503/cmaj.190777 32152052 PMC7062440

[B30] Reed J. E. Howe C. Doyle C. Bell D. (2018). Simple rules for evidence translation in complex systems: a qualitative study. BMC Med. 16, 92. 10.1186/s12916-018-1076-9 29921274 PMC6009041

[B31] Ritchie M. J. Dollar K. M. Kearney L. K. Kirchner J. E. (2014). Research and services partnerships: Responding to needs of clinical operations partners: transferring implementation facilitation knowledge and skills. Psychiatr. Serv. 65, 141–143. 10.1176/appi.ps.201300468 24492898

[B32] Rollins A. L. Salyers M. P. Tsai J. Lydick J. M. (2010). Staff turnover in statewide implementation of ACT: relationship with ACT fidelity and other team characteristics. Adm. Policy Ment. Health 37, 417–426. 10.1007/s10488-009-0257-4 20012481 PMC2888664

[B33] Salyers M. P. Rollins A. L. Kelly Y. F. Lysaker P. H. Williams J. R. (2013). Job Satisfaction and Burnout Among VA and Community Mental Health Workers. Adm. Policy Ment. Health Ment. Health Serv. Res. 40, 69–75. 10.1007/s10488-011-0375-7 21972060 PMC3980458

[B34] Schreiter S. Speerforck S. Schomerus G. Gutwinski S. (2021). Homelessness: care for the most vulnerable – a narrative review of risk factors, health needs, stigma, and intervention strategies. Curr. Opin. Psychiatry 34, 400–404. 10.1097/YCO.0000000000000715 33993170

[B35] Semborski S. Redline B. Madden D. Granger T. Henwood B. (2021). Housing interventions for emerging adults experiencing homelessness: a scoping review. Child. Youth Serv. Rev. 127. 10.1016/j.childyouth.2021.106081 34421161 PMC8372952

[B36] Smelson D. A. Yakovchenko V. Byrne T. Mccullough M. B. Smith J. L. Bruzios K. E.. (2022). Testing implementation facilitation for uptake of an evidence-based psychosocial intervention in VA homeless programs: a hybrid type III trial. PLoS ONE 17, e0265396. 10.1371/journal.pone.0265396 35298514 PMC8929696

[B37] Social Programs that Work (2018). Evidence Summary for the Critical Time Intervention. Available online at: https://evidencebasedprograms.org/document/critical-time-intervention-evidence-summary/ (accessed April 25 2021).

[B38] Stafford A. Wood L. (2017). Tackling Health Disparities for People Who Are Homeless? Start with Social Determinants. Int. J. Environ. Res. Public Health 14, 1535. 10.3390/ijerph14121535 29292758 PMC5750953

[B39] Sullivan W. P. Kondrat D. C. Floyd D. (2015). The Pleasures and Pain of Mental Health Case Management. Soc. Work Ment. Health 13, 349–364. 10.1080/15332985.2014.955942

[B40] Susser E. Valencia E. Conover S. Felix A. Tsai W. Y. Wyatt R. J. (1997). Preventing recurrent homelessness among mentally ill men: a “critical time” intervention after discharge from a shelter. Am. J. Public Health 87, 256–262. 10.2105/AJPH.87.2.256 9103106 PMC1380803

[B41] Tidmarsh G. Whiting R. Thompson J. L. Cumming J. (2022). Assessing the fidelity of delivery style of a mental skills training programme for young people experiencing homelessness. Eval. Program Plann. 94, 102150. 10.1016/j.evalprogplan.2022.102150 35952482

[B42] Tsai J. Rosenheck R. A. (2015). Risk Factors for Homelessness Among US Veterans. Epidemiol. Rev. 37, 177–195. 10.1093/epirev/mxu004 25595171 PMC4521393

[B43] Tsemberis S. Gulcur L. Nakae M. (2004). Housing First, consumer choice, and harm reduction for homeless individuals with a dual diagnosis. Am. J. Public Health 94, 651–656. 10.2105/AJPH.94.4.651 15054020 PMC1448313

[B44] Valenstein-Mah H. Greer N. Mckenzie L. Hansen L. Strom T. Q. Wiltsey Stirman S.. (2020). Effectiveness of training methods for delivery of evidence-based psychotherapies: a systematic review. Implement. Sci. 15, 40. 10.1186/s13012-020-00998-w 32460866 PMC7251851

[B45] Von Thiele Schwarz U. Giannotta F. Neher M. Zetterlund J. Hasson H. (2021). Professionals' management of the fidelity–adaptation dilemma in the use of evidence-based interventions—an intervention study. Implement. Sci. Commun. 2, 31. 10.1186/s43058-021-00131-y 33726864 PMC7962232

[B46] Weightman A. L. Kelson M. J. Thomas I. Mann M. K. Searchfield L. Hannigan B.. (2022). PROTOCOL: Exploring the effect of case management in homelessness per components: A systematic review of effectiveness and implementation, with meta-analysis and thematic synthesis. Campbell Syst. Rev. 18, e1220. 10.1002/cl2.1220 PMC8866910 36908653

[B47] Wiltsey Stirman S. (2022). Implementing evidence-based mental-health treatments: attending to training, fidelity, adaptation, and context. Curr. Dir. Psychol. Sci. 31, 436–442. 10.1177/09637214221109601

[B48] Wiltsey Stirman S. Baumann A. A. Miller C. J. (2019). The FRAME: an expanded framework for reporting adaptations and modifications to evidence-based interventions. Implement. Sci. 14, 58. 10.1186/s13012-019-0898-y 31171014 PMC6554895

[B49] Wirth T. Mette J. Prill J. Harth V. Nienhaus A. (2019). Working conditions, mental health and coping of staff in social work with refugees and homeless individuals: A scoping review. Health Soc. Care Community 27, e257–e269. 10.1111/hsc.12730 30821875 PMC6850100

